# Military Chronic Musculoskeletal Pain and Psychiatric Comorbidity: Is Better Pain Management the Answer?

**DOI:** 10.3390/healthcare4030038

**Published:** 2016-06-30

**Authors:** Cindy A. McGeary, Donald D. McGeary, Jose Moreno, Robert J. Gatchel

**Affiliations:** 1 Department of Psychiatry, The University of Texas Health Science Center at San Antonio, San Antonio, TX 78229, USA; mcgearyc@uthscsa.edu (C.A.M.); morenojl@uthscsa.edu (J.M.); 2 Department of Psychology, The University of Texas at Arlington, Arlington, TX 76019, USA; gatchel@uta.edu

**Keywords:** chronic musculoskeletal pain, low back pain, psychiatric comorbidities, PTSD, depression, military service members

## Abstract

Chronic musculoskeletal pain, such as low back pain, often appears in the presence of psychiatric comorbidities (e.g., depression, posttraumatic stress disorder (PTSD)), especially among U.S. military service members serving in the post-9/11 combat era. Although there has been much speculation about how to best address pain/trauma psychiatric symptom comorbidities, there are little available data to guide practice. The present study sought to examine how pre-treatment depression and PTSD influence outcomes in a functional restoration pain management program using secondary analysis of data from the Department of Defense-funded Functional and Orthopedic Rehabilitation Treatment (FORT) trial. Twenty-eight FORT completers were analyzed using a general linear model exploring how well depression and PTSD symptoms predict post-treatment pain (Visual Analog Scale (VAS) pain rating), disability (Oswestry Disability Index; Million Visual Analog Scale), and functional capacity (Floor-to-Waist and Waist-to-Eye Level progressive isoinertial lifting evaluation scores) in a sample of active duty military members with chronic musculoskeletal pain and comorbid depression or PTSD symptoms. Analysis revealed that pre-treatment depression and PTSD symptoms did not significantly predict rehabilitation outcomes from program completers. Implications of these findings for future research on trauma-related pain comorbidities are discussed.

## 1. Introduction

The comorbidity between chronic musculoskeletal pain conditions (such as low back pain) and psychiatric conditions (like depression and posttraumatic stress disorder (PTSD)) has been well documented in the extant research literature; this is especially salient in studies of U.S. military service members who are at increased risk of developing both musculoskeletal pain and psychiatric trauma symptoms during post-9/11 military service [1,2]. Studies of United States military service members have found rates of depression disorders ranging from 10% to 46% [3,4], and PTSD from 2% to 60% [4]. Psychiatric disorder prevalence has been found to be similar between both active duty and reserve military components [5]. Service members with a chronic musculoskeletal pain condition are likely to develop more than one psychiatric comorbidity, and there is good reason to believe that military members with either PTSD or depression develop a vulnerability to the other condition. For example, a 2007 study of military veterans found that 36% of veterans with depression also screened positive for PTSD [6]. This comorbidity may be due to the overlapping symptoms found in both depression and PTSD. Comorbid depression and PTSD can result in significantly increased risk of suicide in service members with traumatic injuries [7].

Posttraumatic stress disorder and depression are particularly common among individuals with chronic pain conditions. Patients have been found to develop depression secondary to chronic pain [8]. One study of Australian military members found that service members with musculoskeletal pain disorders were at increased risk of mental health-related quality of life (including diagnoses of depression and PTSD) problems [9]. Conversely, individuals with either depression or PTSD are more likely to report severe problems with pain and lower quality-of-life than those without psychiatric conditions [10]. Both PTSD and depression can affect pain in myriad ways. In a randomized controlled trial of 250 veterans, it was found that depression and PTSD had significant independent influence over quality-of-life, psychosocial well-being, and disability in veterans with chronic pain [11]. Some suggest that the comorbidity of PTSD and chronic pain may actually be mediated by depression and facets of anxiety [12] and through feelings of sadness and fear [13], although others suggest that the pain-PTSD comorbidity is mediated through avoidance [14].

Individual treatments for military PTSD, depression, and chronic musculoskeletal pain are well-documented (cf [15]), but there are still fundamental questions about how to best address comorbid pain and psychiatric trauma symptoms. Numerous theories of these comorbidities have been postulated [16], but there is not much evidence to support any of them based on the extant research. Thus, there is little guidance on the best treatment approaches to comorbid trauma pain, and most studies of pain and psychiatric comorbidity emphasize a need for integrated pain and psychosocial intervention to address the complexity of the problem [14,16]. Some small, randomized studies have found promising pain-related outcomes from integrated cognitive and behavioral therapy programs designed to actively address both chronic pain and PTSD [17,18], but there is still not enough evidence to fully support an integrated approach. More information is needed about how PTSD and depression affect functional outcomes in programs designed to specifically address chronic pain (with little to no emphasis on psychiatric comorbidity). Unfortunately, few clinical trials have been developed specifically to meet this purpose, and extant guidance describing the integration between interventions for chronic pain and psychiatric comorbidities are limited “recommendations” without much justification or description for how these services could or should be integrated [16].

The present study is a preliminary attempt to explore the contribution of psychiatric comorbidities to functional pain management outcomes, accomplished through a secondary analysis of the data from the Functional and Orthopedic Rehabilitation Treatment (FORT) pain management program, a randomized clinical trial of military pain management funded by the Department of Defense [19]. The FORT trial was designed to assess the efficacy of a functional restoration pain management program for military service members with chronic pain, but was not designed or powered to specifically assess how psychiatric comorbidities impacted treatment outcomes. However, FORT participants presented with a range of psychiatric comorbidities that were measured as part of the trial and may shed preliminary light on the question of pain and psychiatric comorbidity. In the FORT study, participants were either randomized to treatment-as-usual (TAU) or the FORT program. FORT participants completed a three-week functional restoration based program. Functional restoration programs focus on improving physical functioning through physical reconditioning, psychosocial interventions, and coping skills training. FORT included an interdisciplinary team approach to pain management that included a group-based psychosocial intervention for pain management and coping skills training and a group-based physical therapy program for reconditioning. Over the course of the three weeks, participants attended 12 psychosocial/coping skills group sessions and 12 physical therapy reconditioning group sessions. Treatment-as-usual participants continued to receive military standard of care through their Military Treatment Facility. Participants included U.S. active duty service members with service-related chronic orthopedic pain from all military branches. Basic demographics and outcomes from the FORT study can be found in Gatchel et al. [19]. In preparation for the present secondary analysis, the study team considered the inclusion of both FORT and TAU participant data. The TAU participants demonstrated little significant change in functional outcomes or psychiatric variables from pre- to post-treatment (or in longer-term follow-up; [19]), but the FORT participants demonstrated significant variability. Because the primary aim of this secondary analysis was to assess systematic change in pain outcomes possibly attributable to pre-treatment psychiatric comorbidity, the study team chose to isolate this preliminary analysis to the participants showing the most change in their outcome data: the FORT participants (all of whom completed the FORT program and are hereafter referred to as FORT completers). Furthermore, because the FORT study was not specifically designed to assess the primary aim of this secondary analysis, the study team chose to limit the preliminary analysis to the pre-treatment to post-treatment interval, forgoing analysis of longer-term outcomes that are more likely to be confounded by unforeseen or unassessed variables influencing the contribution of psychiatric comorbidity to long-term outcomes. FORT completer data were analyzed to address the following hypotheses:

H1: Pre-treatment pain severity (based on visual analog pain ratings) and disability (based on functional capacity evaluation and self-report measure of disability) will be significantly related to pre-treatment depression and PTSD symptoms.

H2: Pre-treatment PTSD will have a significant effect on pre- to post-treatment pain and disability outcomes among FORT completers.

H3: Pre-treatment depression will have a significant effect on pre- to post-treatment pain and disability outcomes among FORT completers.

H4: Depression, but not PTSD symptoms will significantly predict post-treatment pain and disability outcomes among FORT completers.

## 2. Materials and Methods

### 2.1. Subjects

This study included a total of 50 active duty military participants spread across all four branches of the military, with a sub-analysis of 28 FORT completers who reported depression and PTSD symptoms at pre-treatment. Details on the FORT intervention can be found in Gatchel et al. [19]. FORT completers were assessed for numerous pain-related psychosocial and physical outcomes. Self-report measures and a functional capacity evaluation were completed at pre-treatment, post-treatment, and 12-month follow-up (although only pre- and post-treatment data are used in the present analysis). Once enrolled, study participants were assigned to one of two treatment groups randomly using a dynamic urn randomization strategy that balanced the groups on age, gender, race/ethnicity, injury site, and time since pain onset. One treatment group received Functional Restoration (FR, *n* = 28) and the other group received treatment-as-usual (TAU, *n* = 22). Review Gatchel et al. for a more in-depth discussion of patient demographics [19].

### 2.2. Measures

Demographic data, including sex, age, branch of service, race/ethnicity, and duration of pain, were collected. Psychosocial and pain measures selected for this study are commonly used psychosocial assessments with strong validity and reliability. The following measures were administered:

Oswestry Disability Inventory (ODI). The ODI is a 10-item self-report questionnaire that measures the degree of experienced functional impairment due to low back pain an individual is experiencing the day of administration [20]. This measure is considered the gold standard for measuring low back pain related disability, with higher scores indicating greater functional impairment.

Million Visual Analog Scale (MVAS). This measure is a 15-item visual analog measure of pain intensity and disability related to low back pain [21]. The MVAS measures current functioning. This instrument produces a total functional disability score ranging from 0 to 150, with higher scores indicating greater pain intensity and disability. The MVAS assesses body functions, daily activities, and social life.

Beck Depression Inventory (BDI-II). The BDI-II is a widely used self-report inventory used to measure depression symptom severity. The BDI-II consists of 21 items assessing symptoms of depression over a two-week period [22].

PTSD CheckList-Military (PCL-M). Since this paper is based on a secondary analysis of the FORT study, the PCL-M was administered rather than the updated PCL-5. The PCL is a 17-question checklist in which participants are asked to endorse symptoms of PTSD commensurate with those listed in the DSM-IV on a Likert-scale, ranging from 1 to 5. The PCL-M has been supported in the literature as a solid PTSD screening instrument, with good correlation of the overall PCL score to the Clinician-Administered PTSD Scale (CAPS) [23].

36-Item Short Form Health Survey Summary (SF-36). The SF-36 is a 36-item self-report questionnaire measuring 8 dimensions (physical functioning, role limitations due to physical problems, pain, general health perceptions, social functioning, vitality, role limits due to emotional health, and mental health) over a four-week period that contribute to 2 summary scales—the Physical Component and Mental Component Summary Scales [24]. It is a measure of health-related quality-of-life, with higher scores indicating greater functioning.

Visual Analog Scale (VAS). The VAS is a nonverbal self-report assessment of pain severity on a 10-cm line [25]. Each end of the line is anchored by an extreme (i.e., no pain or worst imaginable pain). Participants are asked to report how much pain they are experiencing at the time of administration. Higher scores on the line indicate greater pain severity.

Physical Measures. A modified version of the California Functional Capacity Protocol [26] was used to evaluate human performance, including functional strength using a progressive isoinertial lifting evaluation [27]. This protocol was administered by a physical therapist using standardized tasks. Lifting measures included a weighted box lift from Floor to Waist (FW) and Waist to Eye-Level (WEL).

### 2.3. Procedures

The FORT study obtained IRB approval from the University of Texas at Arlington and Wilford Hall Medical Center located on Lackland Air Force Base. All applicable ethical standards were followed in accordance with the 1964 Helsinki declaration. The FORT program was a Department of Defense (DoD)-funded study that included an intensive three-week interdisciplinary chronic pain management intervention based on a functional restoration pain model (described above). The intervention included group-based physical therapy, and cognitive-behavioral group therapy focused on increasing overall functioning. Once consent was obtained, participants were randomly assigned to one of two conditions. The treatment conditions consisted of FR treatment or TAU. Study participants were assessed for physical and psychosocial variables associated with chronic pain experience at pretreatment, post-treatment, and one-year follow-up, although only the pre- and post-treatment assessments for FORT completers who reported pre-treatment depression and PTSD symptoms were used in the present analysis.

### 2.4. Data Analysis

The contribution of psychiatric conditions (depression and PTSD) to post-treatment pain and disability were assessed using general linear models (GLM). To prepare for GLM, pain, disability and psychiatric data were scrutinized for normality to ensure that underlying assumptions of GLM were met. Assessment of linear relationship between the criterion (pain VAS, ODI, MVAS, Lifting) and predictor (depression, PTSD) variables was assessed using a zero-order Pearson product-moment correlation matrix. Relevant demographic variables (gender, age, service branch) were analyzed using ANOVA and correlation (based on data structure) to detect systematic differences in criterion variables that would require inclusion of these variables in GLM as covariates. Pre-treatment scores for the criterion variables were included in all GLM models as covariates.

## 3. Results

The sample included 28 successful completers of the FORT pain management program who also reported pre-treatment depression and PTSD symptoms. FORT completers were 57% male, and all of the completers were serving in either the United States Air Force (USAF; 82%) or the United States Army (USA; 18%). Approximately 80% of the FORT completers presented with spinal pain as their primary complaint, with low back pain representing the majority of spinal pain cases. Over half of the sample self-identified as Caucasian, non-Hispanic. The average age of the sample was 36 years old, with an average time in pain of 61 months. There were no significant differences on any demographic or pain outcome variables between the 28 FORT completers and TAU participants, although FORT completers did report significantly lower depression symptoms at pre-treatment than TAU participants (see Table 1).

As shown in Table 2, FORT completers demonstrated a significant correlation between PTSD symptom scores, and both self-report disability and a measure of functional capacity at pre-treatment. Self-report disability was positively correlated with PTSD on the ODI and MVAS measures, accounting for 11% to 28% of the variance in these measures. PTSD symptoms were negatively correlated with waist-to-eye level lifting, but did not demonstrate a significant relationship with floor-to-waist lifting. Depression scores demonstrated a similar pattern. Pre-treatment BDI scores were significantly and positively related to pre-treatment ODI and MVAS scores, accounting for 8% to 26% of the variance based on the coefficient of determination. Once again, depression symptoms were negatively correlated with pre-treatment waist-to-eye level lifting, but were not significantly related to floor-to-waist level lifting. Neither psychiatric symptom was significantly correlated with pain intensity rating.

PTSD and depression scores were entered into a general linear model (GLM) to examine the extent to which pre-treatment psychiatric symptom scores predict pain management outcomes (including self-report disability, functional capacity, and pain severity). One-way ANOVA and Pearson product-moment correlation were used to identify potential covariates for entry into the GLM. There was a significant difference between the sexes on floor-to-waist and waist-to-eye level lifting at post-treatment (with males lifting more than females), and a significant difference between the military services on ODI score (with US Army members reporting more disability than Air Force personnel). Sex was entered as a covariate in analysis of functional capacity variables, and service branch was entered as a covariate in analysis of ODI scores. As shown in Table 3, there were no significant effects of depression and PTSD symptoms on any of the criterion variables after controlling for pre-treatment scores and identified covariates, although psychiatric symptoms reached near-significance on a few criteria. PTSD symptoms (as measured by the PCL-M) had a near-significant association with post-treatment MVAS self-report disability scores after controlling for pre-treatment MVAS scores (*p* = 0.058) and depression symptoms, and PTSD symptoms had a near-significant association with post-treatment waist-to-eye level lifting (*p* = 0.077) after controlling for pre-treatment lifting capacity and gender.

Because neither psychiatric symptom variable was able to significantly predict posttreatment pain outcomes on its own, analyses of Hypothesis 4 (evaluating an interaction between the two symptoms) were not conducted.

## 4. Discussion

The present study is the first to explore the effect of common comorbid psychiatric symptoms on post-treatment outcomes for chronic musculoskeletal active duty pain patients who completed a functional restoration program. As expected, this secondary analysis revealed that pre-treatment scores of pain severity and disability were found to be related to pre-treatment depression and PTSD symptoms. This echoes numerous extant findings emphasizing the significant relationship between chronic pain symptoms and psychiatric comorbidities. Surprisingly, despite of the significant pre-treatment correlations between depression and PTSD symptoms with pain-related objective and subjective disability measures, neither had a significant influence on post-treatment functional outcomes in this military cohort. Although there have been numerous recommendations for integrating pain management and psychosocial management of comorbid pain and depression/PTSD to effectively address the comorbidity, the present findings suggest that it may be possible to effectively address comorbid chronic pain without specifically attending to the psychiatric symptoms (at least for those with subsyndromal psychiatric symptoms). One potential explanation for the present finding is that the FORT program led to a reduction in psychiatric symptoms, lessening their effect on post-treatment functional outcomes. This hypothesis makes sense in light of the significant correlations between depression/PTSD symptoms and functional/disability measures at pre-treatment, as well as evidence from previously published findings from this cohort showing a significant improvement in psychiatric symptoms from pre- to post-treatment among treatment completers [19]. It is also possible, however, that effectively reducing pain-related disability and functional capacity improved depression and PTSD symptoms based on links between these symptoms and chronic pain that are mediated by disability and functional incapacity. Indeed, depression and PTSD have been shown in prior studies to have a strong influence on disability and functioning that lead to a more intense pain experience [28], although other studies have shown that pain outcomes can improve even when psychiatric symptoms do not. For example, a study of 142 non-severe head-injured trauma patients found that those who completed a multidimensional pain management program reported no improvement in their psychiatric symptoms, but did report significant pain relief at post-treatment [29]. This aligns with other evidence in the extant literature showing that the presence of chronic pain does little to predict treatment outcomes in psychosocial interventions targeting PTSD [30]. Clearly, more work is needed to explore the relationship between these conditions.

There were several limitations of this research study. First, the present re-analysis examined a small small sub-sample (*n* = 28) of FORT data over a brief time (3 weeks). This was done to focus the analysis on a more variable data sub-sample most likely to illuminate the influence of pre-treatment psychiatric symptoms on functional rehabilitation outcomes, but the narrow sample likely diminished statistical power and obscured some significant findings (although some near-significant relationships were still uncovered). A large majority of the sample was Air Force (77%); therefore, results may not be generalizable to the other services. It would be beneficial in the future to have a broader representation of the services included in the FORT program. This study was conducted prior to military members returning from Operation Enduring Freedom (OEF) and Operation Iraqi Freedom (OIF), so reported psychiatric comorbidities for the present sample were likely milder than those of more recent veterans. However, the preliminary data generated by this research offers a good foundation for future research because musculoskeletal injuries increase among military service members and veterans due to injuries related to OEF/OIF. In fact, 47% of OEF/OIF returning deployers are reporting chronic pain [5], and chronic pain is likely to continue to grow even as rates of PTSD and traumatic brain injury stabilize in the U.S. military and Veterans Affairs [31]. Further research in functional restoration is needed with returning deployers based on increased musculoskeletal pain disorders in the military.

## 5. Conclusions

This study raises continued questions regarding the best treatment for complex chronic musculoskeletal pain in military and veteran populations, and gives some rise to questions about the need for integrated psychiatric and pain management interventions to adequately address complex polymorbid pain. Although this preliminary re-analysis did not definitively answer the research question, it did offer an early finding supporting the potential of functional improvements alone as adequate for good pain management is this complex population. Despite multiple calls for integrated treatment programs (which can be expensive and difficult to implement due to the specialty resources required [32]), it is possible that the emphasis of future study and research should be on finding effective pain management interventions for this complex population, ignoring interventions that are effective for the amelioration of comorbid psychiatric symptoms. Future studies should also explore the long-term socioeconomic and quality-of-life implications of improved function and disability outcomes in complex comorbid pain patients. If improvements in function and disability lead to decreased healthcare utilization and increased quality-of-life in military comorbid pain patients (as has already been demonstrated in some preliminary studies [33,34,35]), then better pain management strategies should certainly receive greater attention.

## Figures and Tables

**Table 1:** Comparison of pre-treatment demographic, pain and psychiatric variables between Functional and Orthopedic Rehabilitation Treatment (FORT) completers and non-completers (who were given treatment as usual).

Variable	Assessment	FORT Completers *N* = 28	TAU *N* = 22	*p*-Value
Demographics	Age (yrs)	36.3	35.8	0.780
	Time in Pain (mos)	61.5	64.1	0.879
	Sex (% male)	57	43	0.890
	Service Branch (% USAF)	82	55	0.064
	Race (% Caucasian, non-Hispanic)	64	64	0.587
Pain Characteristics	Self-Report Disability (Mean (SD))			
	MVAS	74.1 (25.3)	78.0 (20.8)	0.586
	ODI	17.2 (8.9)	18.7 (6.1)	0.488
	Functional Capacity (Mean (SD))			
	Floor-to-Waist Lift (lbs)	49.3 (36.5)	46.7 (20.8)	0.779
	Waist-to-Eye Lift (lbs)	42.4 (15.9)	36.0 (14.3)	0.144
	Health-Related Quality of Life (Mean (SD))			
	SF-36 Physical	34.4 (10.4)	35.8 (6.9)	0.555
	SF-36 Mental	51.0 (8.7)	50.7 (8.8)	0.887
	Pain Intensity (Mean (SD))			
	VAS	5.6 (3.6)	4.8 (4.8)	0.498
Psychiatric Symptoms	Depression (Mean (SD))			
	BDI-2	9.5 (6.9)	14.9 (10.2)	0.034
	PTSD (Mean (SD))			
	PCL-M	27.9 (8.3)	31.9 (11.6)	0.164

**Table 2:** Correlation of Posttraumatic stress disorder CheckList-Military (PCL-M) and Beck Depression Inventory (BDI) scores to self-report measures of disability (Oswestry Disability Inventory (ODI), Million Visual Analog Scale (MVAS)) and lifting scores (Floor-to-Waist (FW), Waist-to-Eye Level (WEL)) among FORT completers.

r (On-Tailed) *p-*Value	ODI	MVAS	FW	WEL	VAS
PCL-M	0.527 *<0.001*	0.326 *0.011*	−0.083 *0.284*	−0.324 *0.011*	0.226 0.057
BDI	0.513 *<0.001*	0.278 *0.025*	−0.058 *0.345*	−0.323 *0.011*	−0.048 0.371

**Table 3:** General linear model (GLM) of PTSD predicting post-treatment self-report disability (controlling for pre-treatment disability scores).

Predictor	Assessment	F-Test	*p*-Value
Pre-Treatment BDI	ODI	1.375	0.264
	MVAS	0.056	0.814
	FW	0.810	0.452
	WEL	1.310	0.281
	VAS	1.210	0.277
Pre-Treatment PCL-M	ODI	1.943	0.137
	MVAS	3.770	0.058
	FW	0.326	0.724
	WEL	2.732	0.077
	VAS	0.186	0.669
